# Hyperventilation Syndrome in a Child: Electrolyte Disturbances and Cardiac Involvement in Anxiety-Related Presentations

**DOI:** 10.3390/pediatric17050087

**Published:** 2025-08-29

**Authors:** Andrea Calandrino, Anna Carla Defilippi, Gemma Eftimiadi, Luca Antonio Ramenghi, Diego Minghetti

**Affiliations:** 1 Neonatal Intensive Care Unit, Department of Maternal and Neonatal Health, IRCCS Istituto Giannina Gaslini, 16147 Genoa, Italy; lucaramenghi@gaslini.org; 2 Pediatric and Neonatology Spoke Unit of Imperia-Sanremo, Department of Maternal and Neonatal Health, IRCCS Giannina Gaslini Institute, 18100 Imperia, Italy; annacarladefilippi@gaslini.org (A.C.D.); gemmaeftimiadi@gaslini.org (G.E.); diegominghetti@gaslini.org (D.M.); 3 Department of Neuroscience, Rehabilitation, Ophtalmology, Genetics, Maternal and Child Health, University of Genoa, 16132 Genoa, Italy

**Keywords:** hyperventilation syndrome, anxiety disorders, hypocalcemia, QTc prolongation

## Abstract

**Background:** Hyperventilation Syndrome (HVS) is a well-recognized physiological consequence of acute anxiety, often resulting in respiratory alkalosis and subsequent electrolyte imbalances. Among these, a reduction in ionized calcium levels can lead to neuromuscular irritability and electrocardiographic abnormalities such as QTc prolongation. Although well-documented in specific settings, including autism spectrum disorders and drug-induced crises, such complications are rarely described in otherwise healthy pediatric patients presenting with isolated anxiety episodes. This report aims to raise awareness of anxiety-driven somatic manifestations, particularly in the context of the rising prevalence of mental health disorders among children and adolescents. **Methods:** We report the case of a previously healthy 10-year-old girl presenting to the emergency department with acute agitation and hyperventilation. Clinical examination revealed neuromuscular symptoms, including Trousseau’s sign and flexion posture. Initial laboratory testing and arterial blood gas analysis indicated respiratory alkalosis with decreased ionized calcium levels, and a resting ECG showed QTc prolongation (510 ms). Treatment included intravenous midazolam, a balanced electrolyte solution, and oral bromazepam during intensive observation with cardiac monitoring. **Results:** The patient’s symptoms progressively improved following anxiolytic and supportive therapy. Electrolyte abnormalities normalized within 48 h, with complete resolution of the prolonged QTc (430 ms). No arrhythmias or other complications occurred. Outpatient psychological follow-up was arranged upon discharge. **Conclusions:** This case underscores the importance of considering anxiety as a primary etiology in pediatric patients with apparent metabolic or cardiac abnormalities. Early psychiatric recognition and targeted supportive care can prevent overtreatment and reduce the burden on emergency and cardiologic resources.

## 1. Introduction

Hyperventilation Syndrome (HVS) is a clinical condition characterized by inappropriate alveolar hyperventilation, leading to a decrease in arterial carbon dioxide tension (PaCO_2_) and a state of respiratory alkalosis [1]. This alkalotic shift induces several ionic and biochemical changes, including a reduction in ionized calcium levels due to increased binding of calcium to serum albumin, as well as intracellular shifts of potassium and phosphate [2,3]. These electrolyte disturbances may produce a variety of symptoms ranging from mild paresthesias to severe neuromuscular irritability, tetany, and even potentially dangerous cardiac arrhythmias [4].

Anxiety and panic disorders are common precipitants of HVS [5]. In some patients, the intense sympathetic activation associated with anxiety can trigger a hyperventilation crisis, exacerbating respiratory alkalosis and its metabolic consequences [6,7]. Among these, hypocalcemia has been recognized as a significant contributor to both neuromuscular and cardiac manifestations, including QTc prolongation, which may predispose patients to life-threatening arrhythmias [8].

Although cases of hypocalcemia secondary to hyperventilation are documented in various clinical settings, including autism spectrum disorders [9] and substance-induced states [10], reports remain limited on presentations involving isolated anxiety-induced hyperventilation and electrocardiographic changes.

We present the case of a child who arrived at the emergency department with anxiety-driven hyperventilation, complicated by ionized hypocalcemia and QTc prolongation.

## 2. Case Report

A previously healthy 10-year-old girl presented to the emergency department in an evident state of agitation. Her past medical history was unremarkable, with no known comorbidities or chronic conditions. However, her parents reported prior isolated episodes of agitation and emotional outbursts, which had spontaneously resolved without requiring medical intervention or pharmacological therapy.

At presentation, the patient was markedly anxious and restless. Physical examination revealed a positive Trousseau’s sign in both hands and a transient equinus posture of the feet, findings consistent with neuromuscular irritability related to hypocalcemia, rather than spasticity. No focal neurological deficits were detected; muscle strength and coordination were preserved, and cranial nerve examination was normal. There was no evidence of altered mental status beyond the marked agitation.

Vital signs at ER admission were as follows: heart rate, 132 beats per minute; respiratory rate, 36 breaths per minute; oxygen saturation, 99% on room air; and blood pressure, 110/65 mmHg. Despite the hyperventilation and visible psychomotor agitation, the patient maintained adequate oxygenation throughout the whole episode.

Laboratory workup, as extensively reported in Table 1, revealed marked respiratory alkalosis, ionized calcium was decreased (1.02 mmol/L), while total serum calcium remained within the normal range.

The remaining routine laboratory tests, including complete blood count, C-reactive protein, liver function tests, and renal function parameters, were all within normal limits.

**Figure 1:**
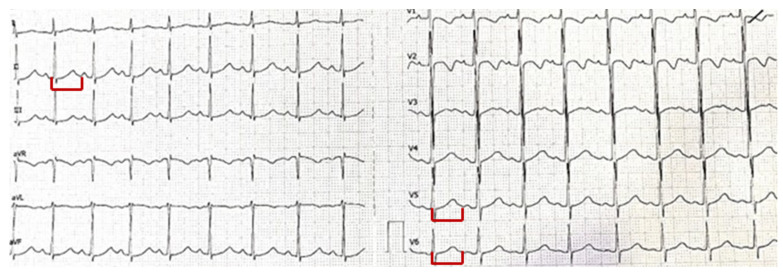
Resting 12-lead ECG at presentation showing sinus rhythm with a prolonged QT interval (labelled in red in DII, V5, and V6). The QT and corrected QT (QTc) intervals are labeled for clarity. QTc was calculated using Bazett’s correction formula and measured 510 ms at admission.

A resting electrocardiogram (ECG) was performed (Figure 1), showing sinus rhythm with a corrected QT interval (QTc) of 510 ms (normal QTc: <460 ms for female children [11]). No other conduction abnormalities or repolarization changes were observed.

The patient was initially treated with mild sedation using intravenous midazolam at a dose of 0.1 mg/kg upon diagnosis. Intravenous hydration with balanced electrolyte solution was also administered to support metabolic stability and correct electrolyte disturbances. She was subsequently admitted to intensive observation with continuous cardiac monitoring for 48 h. During this period, anxiolytic therapy was continued with oral bromazepam 2.5 mg/mL at a dosage of 10 drops once daily on the second and third day.

Oral bromazepam was chosen due to its availability in drop formulation in our local formulary and its favorable anxiolytic profile for short-term use. While lorazepam and diazepam are more commonly used in pediatric emergency protocols, bromazepam was considered appropriate in this setting for its ease of titration, mild sedative effect, and favorable tolerability in our clinical experience.

Progressive resolution of anxiety symptoms was observed, accompanied by normalization of vital signs, arterial blood gas parameters, and serum electrolyte levels.

Serial electrocardiographic monitoring demonstrated a reduction of the QTc interval, which returned to a normal value of 430 ms. After clinical stabilization, the patient was discharged home with the recommendation to undergo a follow-up ECG evaluation 10 days later. In addition, the patient was referred for outpatient psychological follow-up after discharge to assess for possible anxiety spectrum disorders; no further information is available regarding the subsequent psychiatric evaluation or long-term management.

## 3. Discussion

In recent years, particularly in the aftermath of the COVID-19 pandemic, a growing body of evidence has documented a marked increase in the prevalence of anxiety disorders and psychiatric conditions among adolescents and young adults [12]. Global health organizations and epidemiological studies have consistently highlighted how the pandemic catalyzed the exacerbation of mental health issues in youth [13,14]. Prolonged social isolation, disruption of school routines, increased academic pressure following remote learning periods, reduced peer interactions, increased family stress, and the pervasive uncertainty surrounding the pandemic have all contributed to creating a vulnerable psychological landscape for adolescents [15,16]. Additionally, greater exposure to digital media, often characterized by alarming or polarizing content, may have further amplified anxiety levels in susceptible individuals [17].

Late childhood and adolescence represent a critical neurodevelopmental window during which the interplay between biological maturation, hormonal fluctuations, and emerging psychosocial challenges predisposes individuals to heightened emotional reactivity [18]. In this context, anxiety may not only manifest as psychological distress but also present with prominent somatic and autonomic features that may be the leading cause of medical evaluation, particularly in acute care settings [19,20,21]. Among these, hyperventilation stands out as a frequent physiological response to acute anxiety episodes [22].

HVS results from excessive breathing, leading to the inappropriate elimination of carbon dioxide and subsequent respiratory alkalosis [1]. The biochemical consequence of this alkalosis is a complex shift in the distribution of plasma ions: the rise in pH enhances the binding affinity of albumin for calcium, leading to a rapid reduction in ionized calcium levels [2]. In contrast, total serum calcium may remain within normal limits. This transient hypocalcemia is directly responsible for the neuromuscular irritability commonly observed, including paresthesias, muscle cramps, Trousseau’s sign, and in some cases, tetany or dystonic posturing, as seen in our patient [4]. Furthermore, hypocalcemia and alkalosis also affect cardiac membrane stability, predisposing patients to electrocardiographic abnormalities, most notably QTc interval prolongation, which was documented in our case [8].

QTc prolongation in the setting of electrolyte imbalance represents a potentially serious arrhythmogenic substrate [23]. However, in our patient, despite a QTc interval reaching 510 ms, no malignant ventricular arrhythmias, syncope, or hemodynamic instability were observed. The absence of underlying structural or electrical heart disease, as reported in the patient history, combined with the rapid reversibility of the biochemical abnormalities, suggests that the arrhythmic risk in these acute anxiety-induced episodes may be self-limiting.

In approaching the QTc prolongation observed in our patient, a brief differential diagnosis was considered. Congenital long QT syndrome (LQTS) was deemed unlikely given the absence of a family history of sudden cardiac death, arrhythmias, or syncope, and the normalization of QTc within 48 h, together with the lack of temporal and spatial dispersion of depolarisation [24]. Drug-induced QT prolongation was excluded since the patient was not on any chronic pharmacologic treatment and no QT-prolonging agents had been administered before presentation. The normal levels of potassium and magnesium ruled out electrolyte disturbances beyond the documented hypocalcemia. Finally, structural cardiac disease was considered unlikely given a normal clinical examination, stable hemodynamics, and absence of ECG features suggestive of conduction abnormalities [25]. This stepwise evaluation supports acute anxiety-related hyperventilation as the most plausible cause of the observed abnormalities.

Considering the abovementioned evaluation, although the QTc interval exceeded 500 ms at presentation, no additional cardiologic investigations such as echocardiography or genetic testing were performed during the acute episode. However, outpatient follow-up was arranged to confirm QTc normalization and to monitor for any recurrence, with the plan to pursue further evaluation if clinically indicated.

Beyond the immediate clinical implications, the present case offers novel insights compared to previously reported instances of hyperventilation-associated hypocalcemia and QTc prolongation. While such complications have been described in association with neurological comorbidities, substance use, or chronic psychiatric conditions [9,10], our patient was a previously healthy 10-year-old experiencing an isolated anxiety episode.

Notably, a pediatric study of 25 adolescents (median age 14 years) undergoing controlled hyperventilation demonstrated an average QTc increase of approximately 35 ms, with some children developing transient repolarization abnormalities, specifically, QTc prolongation even in the absence of underlying LQTS, suggesting that hyperventilation alone can mimic pathological prolongation in healthy children [26,27,28]. Furthermore, a long-term follow-up of 34 pediatric cases revealed that many healthy children presenting with hyperventilation syndrome went on to exhibit persistent anxiety symptoms into adulthood, despite no antecedent neurological or metabolic disease [29].

Compared to these reports, our case is unique in involving a previously healthy 10-year-old girl whose acute anxiety-induced hyperventilation led to noticeable ionized hypocalcemia and QTc prolongation that resolved rapidly with supportive care. This reinforces that even in younger pediatric age groups and in the absence of comorbidities, somatic manifestations of anxiety can include significant metabolic and ECG abnormalities.

The main point of treatment in such presentations remains the prompt recognition of the underlying anxiety-driven hyperventilation and its physiological consequences [7]. In our case, no specific cardiologic intervention or antiarrhythmic therapy was required. Supportive care focused on reducing anxiety through reassurance, environmental de-escalation, and low-dose anxiolytic medication, in addition to the administration of intravenous electrolytes, resulted in rapid symptom resolution and normalization of the QTc interval. This emphasizes the crucial role of targeting the primary psychiatric trigger rather than pursuing extensive cardiologic or neurologic workup in the absence of other concerning features [20].

Although formal pediatric guidelines dedicated solely to anxiety-related hyperventilation syndrome are scarce, available pediatric-focused literature, including long-term series evaluating hyperventilation syndrome in children, supports a management strategy involving prompt recognition, reassurance, and minimal unnecessary workup [28,29]. These studies underscore that many affected children are otherwise healthy and that anxiety-triggered hyperventilation responded well to supportive care. A recent pediatric respiratory review further recommends a multidimensional management approach combining breathing retraining, psychological support, and adjunctive therapies when needed [30]. This framework aligns with our clinical approach and reinforces the need for early psychological referral and long-term follow-up.

Importantly, this case underscores the growing need for heightened awareness among clinicians, particularly in emergency and pediatric care settings, regarding the somatic presentations of psychiatric disorders in adolescents [19]. Failure to recognize the psychiatric underpinning may lead to unnecessary diagnostic procedures, hospitalization, and potential overtreatment, further amplifying the distress for both patients and families [31]. The generalizability of this case is further reinforced by the increasing prevalence of anxiety disorders in children and adolescents, especially in the post-pandemic era, making similar presentations more likely to be encountered in clinical practice.

## 4. Conclusions

Clinicians should be aware that acute anxiety in children and adolescents can manifest with significant biochemical and cardiac alterations, even in the absence of underlying organic disease. Hyperventilation-induced hypocalcemia and QTc prolongation, while alarming, may resolve rapidly with anxiolysis and supportive care. Recognizing the psychiatric origin early is essential to avoid unnecessary investigations and to prioritize psychological support as part of the treatment plan. Integrating mental health evaluation into acute pediatric care is no longer optional; it is essential.

## Figures and Tables

**Table 1:** Arterial Blood Gas Analysis and Electrolyte Panel at Admission.

Parameter	Measured Value	Reference Range
Arterial Blood Gas		
pH	7.60	7.35–7.45
PaCO_2_ (mmHg)	22	35–45
PaO_2_ (mmHg)	98	80–100
HCO_3_^−^ (mmol/L)	24	22–26
Base Excess (mmol/L)	+3	−2–+2
SaO_2_ (%)	99%	95–100%
Serum Electrolytes		
Sodium (mmol/L)	138	135–145
Potassium (mmol/L)	3.8	3.5–5.0
Chloride (mmol/L)	98	98–106
Serum Calcium (mg/dL)	9.3	8.5–10.5
Ionized Calcium (mmol/L)	1.02	1.15–1.33
Magnesium (mmol/L)	0.82	0.70–1.00

## Data Availability

The datasets generated during and/or analyzed during the current study are not publicly available but are available from the corresponding author on reasonable request.

## Abbreviations

The following abbreviations are used in this manuscript:

HVS	Hyperventilation Syndrome
QTc	Corrected QT Interval
ECG	Electrocardiogram
ER	Emergency Room
LQTS	Long QT Syndrome
